# Comparing a porphyrin- and a coumarin-based dye adsorbed on NiO(001)

**DOI:** 10.3762/bjnano.10.88

**Published:** 2019-04-15

**Authors:** Sara Freund, Antoine Hinaut, Nathalie Marinakis, Edwin C Constable, Ernst Meyer, Catherine E Housecroft, Thilo Glatzel

**Affiliations:** 1 Department of Physics, University of Basel, Klingelbergstrasse 82, 4056 Basel, Switzerland; 2 Department of Chemistry, University of Basel, BPR 1096, Mattenstrasse 24a, 4058 Basel, Switzerland

**Keywords:** coumarin, Kelvin probe force microscopy, metal oxide, molecular resolution, nickel oxide (NiO), non-contact atomic force microscopy, porphyrin

## Abstract

Properties of metal oxides, such as optical absorption, can be influenced through the sensitization with molecular species that absorb visible light. Molecular/solid interfaces of this kind are particularly suited for the development and design of emerging hybrid technologies such as dye-sensitized solar cells. A key optimization parameter for such devices is the choice of the compounds in order to control the direction and the intensity of charge transfer across the interface. Here, the deposition of two different molecular dyes, porphyrin and coumarin, as single-layered islands on a NiO(001) single crystal surface have been studied by means of non-contact atomic force microscopy at room temperature. Comparison of both island types reveals different adsorption and packing of each dye, as well as an opposite charge-transfer direction, which has been quantified by Kelvin probe force microscopy measurements.

## Introduction

**Figure 1:**
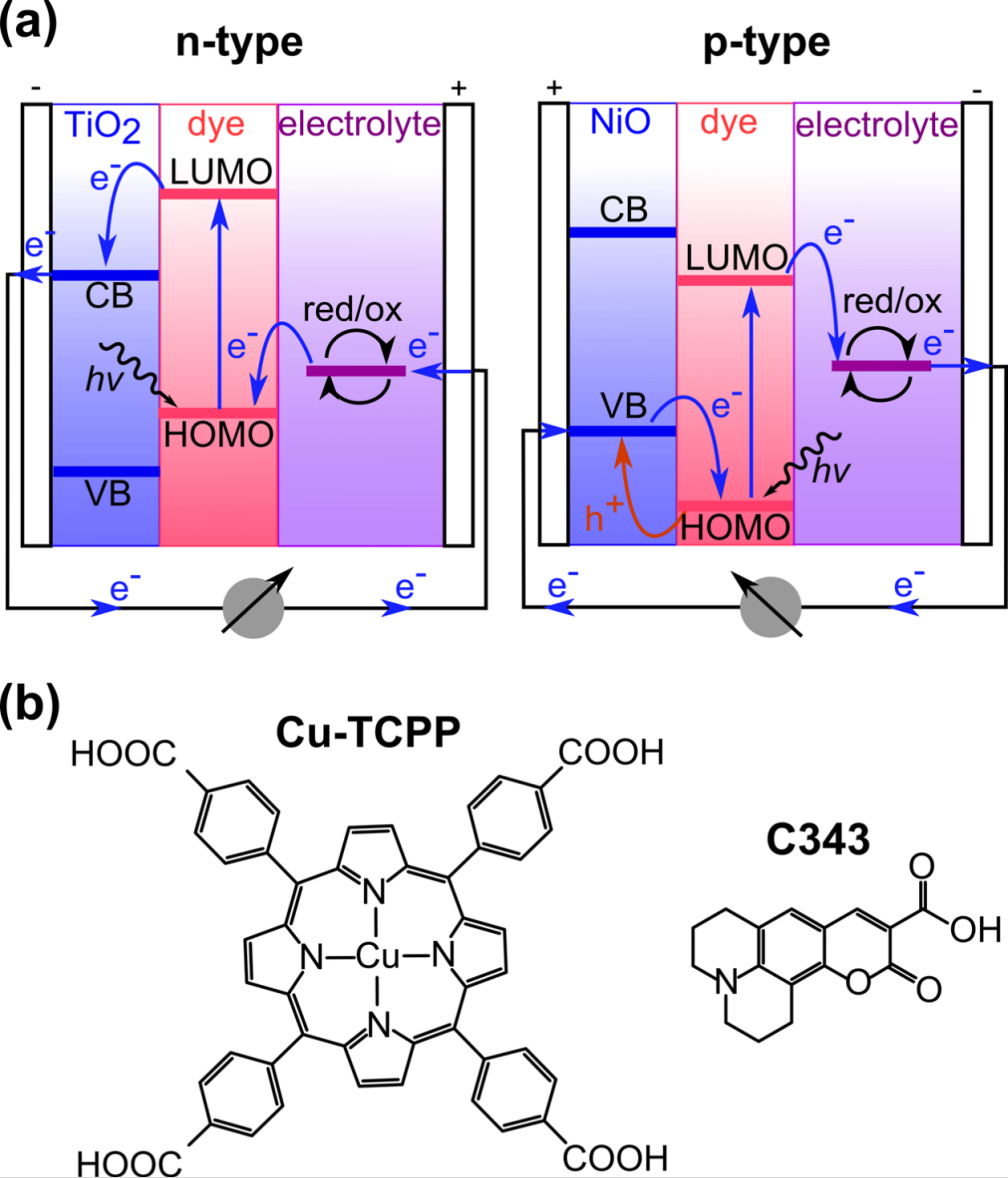
(a) Principle of n- and p-type DSSCs showing opposite charge transfer directions. (b) Structures of Cu-TCPP and C343.

With regard to its use in dye-sensitized solar cells (DSSCs), the wide-bandgap n-type semiconductor TiO_2_ has become one of the most extensively studied metal oxides, especially in the context of scanning probe microscopy (SPM) [1]. The working principle of an n-type DSSC, which is shown in Figure 1a, relies on the functionalization of TiO_2_ surfaces with dye molecules enabling the absorption of light in the visible region of the sun spectrum. The photons are absorbed by the dye molecules leading to the excitation of an electron from the highest occupied molecular orbital (HOMO) to the lowest unoccupied molecular orbital (LUMO) of the dye and subsequent injection into the conduction band (CB) of the semiconductor [2]. This charge transfer, which occurs from the dye molecules towards the surface of the semiconductor, offers the possibility of designing specific hybrid devices with photoactive anodes consisting of functionalized TiO_2_.

In contrast to TiO_2_ [3–11], wide-bandgap p-type semiconductors, such as NiO, and their functionalization with sensitizers, have been less extensively studied by using SPM [12–15]. NiO was the first reported p-type wide-bandgap semiconductor [16], and can be used for the fabrication of p-type DSSCs with photoactive cathodes, a first step towards the design of tandem solar cells with two photoactive electrodes [17–18]. In p-type DSSCs, the charge transfer is in the opposite direction to that in n-type devices. Holes are injected from the highest occupied molecular orbital (HOMO) of the dye to the valence band (VB) of the semiconductor after photon absorption [17,19–20], resulting in an electron transfer from the surface of the semiconductor towards the dyes (see Figure 1a).

In other terms, the direction of charge transfer relies on the electron affinity of the dyes and on their HOMO and LUMO levels compared to the CB and VB of the semiconductor. Typically, dyes are specifically designed to be compatible with one or the other type of device. Consequently, the careful choice of the dye is crucial for the optimal function of n- or p-type DSSCs. Because of its electron-donor character, copper(II) *meso*-tetrakis(4-carboxyphenyl)porphyrin (Cu-TCPP) has been studied for the fabrication of n-type DSSCs [21–22]. In contrast, Coumarin 343 (C343) is an electron acceptor and is used for the design of p-type devices [23–24]. Both molecules structures are shown in Figure 1b.

In this paper, non-contact atomic force microscopy (nc-AFM) is used at room temperature (RT) to compare the properties of the two dyes deposited on a NiO(001) single-crystal surface. Under ultrahigh vacuum (UHV) conditions, the adsorption modes of both molecules on the surface of NiO(001) are studied and their charge state upon adsorption are investigated by Kelvin probe force microscopy (KPFM) [25]. This technique is used to observe and quantify the contact potential difference (CPD) changes between the metal oxide surface and the molecular layers and to determine the corresponding dipole moments.

## Results and Discussion

**Figure 2:**
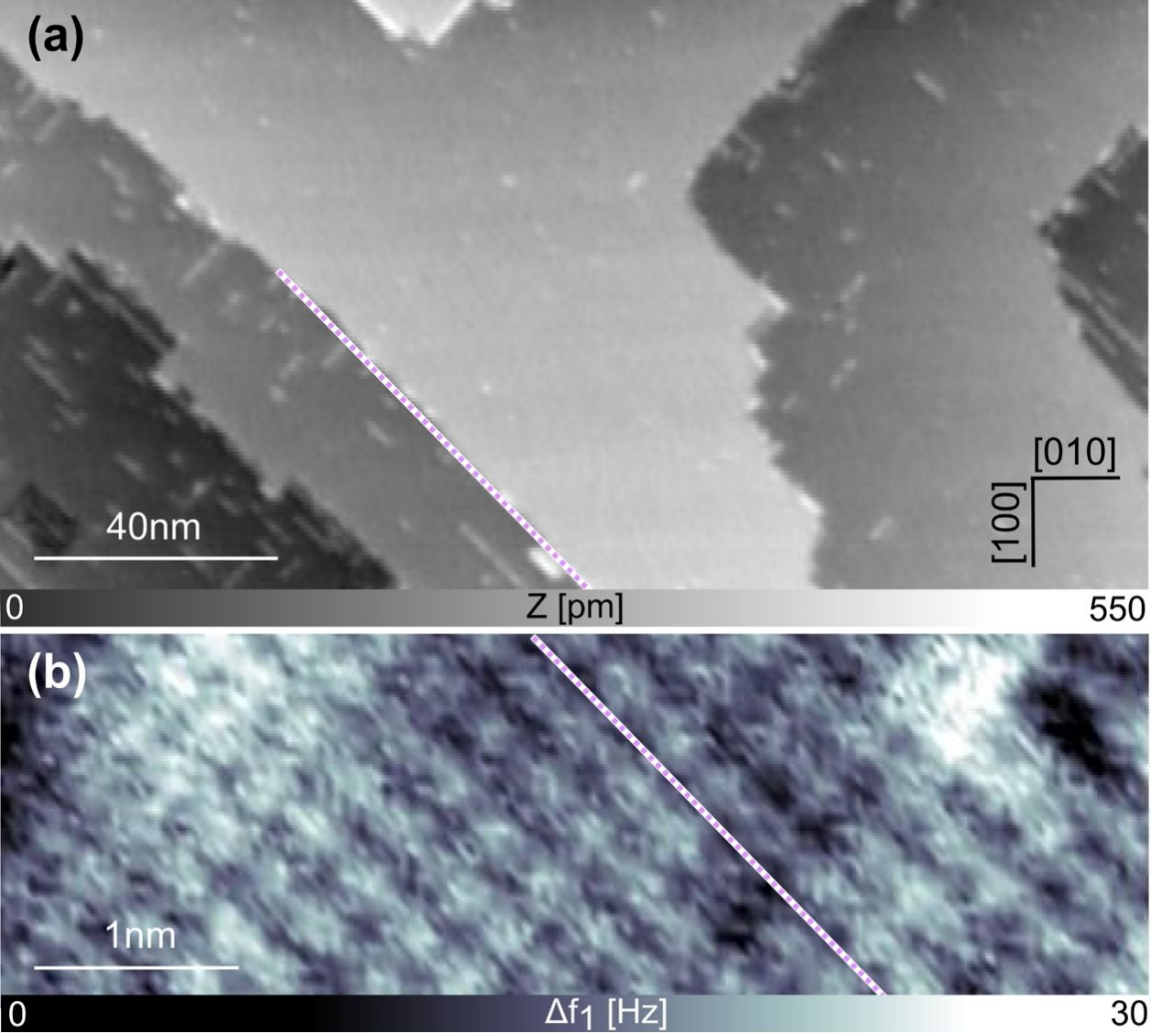
The surface of NiO(001). (a) Large-scale topographic image of the NiO(001) crystal showing clean terraces running along the [110] direction of the surface (scan parameters: 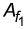 = 4 nm, Δ*f*_1_ = −9 Hz). (b) Frequency-shift (Δ*f*_1_) signal of the same surface at atomic resolution, recorded in the second line scan of the multipass technique with following scan parameters: 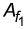 = 4 nm, Δ*f*_1_ = −42 Hz) and *z*_offset_ = −700 pm.

Atomically clean NiO surfaces, mandatory for reliable SPM studies, are difficult to prepare because of the hardness of the material and its high reactivity [12–15,26–34]. Figure 2a shows a topographic image measured by nc-AFM of the bare NiO(001) surface that was prepared by in situ cleavage and annealing. The surface shows extended terraces separated by monoatomic steps that are 210 pm in height. Additionally, some line-shaped defects are observed all over the terraces. These defects, thought to be due to segregation of bulk impurities, were not further investigated in the present study and do not influence the reported results.

Figure 2b shows the frequency-shift signal acquired using the multipass imaging technique [14–15,35–36] clearly showing atomic resolution of the NiO(001) surface. Employing this method, the crystallographic directions of the substrate are resolved with atomic accuracy, and upon comparing them with the large-scale image, one can deduce that the step edges of NiO(001) as well as the observed defects run along the [110] direction (see violet dotted lines in Figure 2). With this physical image of the atomically resolved structure of the clean NiO(001) surface in mind, the adsorption properties of the Cu-TCPP and the C343 dye molecules were investigated.

### Cu-TCPP islands formed on NiO(001)

**Figure 3:**
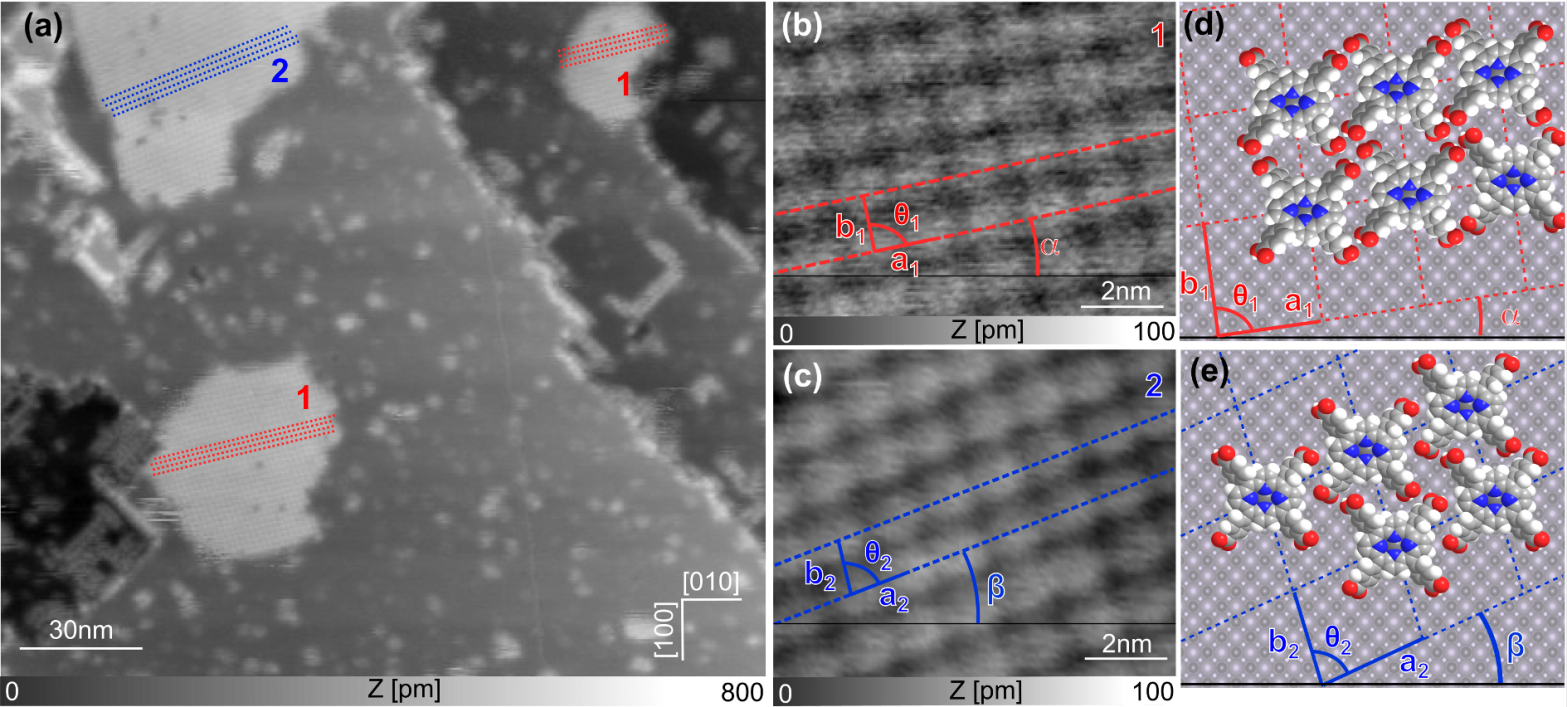
(a) Large-scale topographic image showing that Cu-TCPP molecules form islands on the surface of NiO(001) (scan parameters: 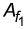 = 7 nm, Δ*f*_1_ = −7 Hz). (b, c) Close-up topographic images recorded on a type-1 and a type-2 island, respectively (scan parameters: 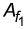 = 4 nm, Δ*f*_1_ = −38 Hz and 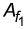 = 7 nm, Δ*f*_1_ = −8 Hz); (d, e) Models corresponding to (b) and (c).

In a first experiment, Cu-TCPP molecules were deposited at RT on a freshly cleaved NiO(001) surface. Figure 3a shows a large-scale topographic image of the molecules adsorbed on the substrate, where it can be seen that Cu-TCPP exhibits either the tendency to aggregate in small clusters at step edges and defects, or to form large molecular islands (up to 70 nm in width). The fact that the island formation takes place at RT indicates a relatively high diffusion rate of the molecules on NiO(001). The emergence of numerous clusters is related to the presence of various defects on the surface that act as anchoring sites for the dyes.

Concentrating on the islands and measuring their heights (250–300 pm), we can conclude that the molecules are lying flat on the substrate. Interestingly, only two types of islands (type 1 and type 2) were found on the sample surface. In both types, molecular rows are aligned with two distinguishable angles (α and β) with respect to the [010] direction of the substrate (see red and blue dotted lines in Figure 3a–c.

Both molecular alignments are shown in more detail in the close-up topographic images displayed in Figure 3b,c recorded on type-1 and type-2 islands, respectively. The angles α and β are measured to be 10 ± 1° and 20 ± 1° with respect to the [010] direction, respectively. The lattice parameters were measured and it was observed that *a*_1_, *b*_1_, *a*_2_ and *b*_2_ are similar in the range of 1.5 ± 0.1 nm, while the angles θ_1_ and θ_2_ of the unit cells clearly differ (88 ± 1° and 82 ± 1°, respectively). The molecular densities of both types are measured to be *D*_1_ = *D*_2_ = 0.46 ± 0.02 nm^−2^.

Based on these high-resolution images and on the measured mesh parameters, the corresponding models in Figure 3d,e can be established. Knowing that Cu-TCPP has a fourfold symmetrical structure with four equivalent anchoring groups, it is assumed, that Cu-TCPP lies flat and is commensurate with the surface of NiO(001). Considering the partial charge distribution of the surface (Ni is δ^+^ and O is δ^−^), it is thought that the metallic core of the molecule is likely to be located above an O atom. In addition, it is expected, that the molecules adopt the same adsorption configuration, independent of the island type, triggered by the formation of H-bonds between the carboxylic groups of adjacent molecules. This results in model values for all the parameters that have been calculated and are presented in Table 1. These values are in very good agreement with the experimental results.

**Table 1:** Experimental values and model parameters of both types of Cu-TCPP islands.

island type	mesh parameters			molecular density	
		
	experiment	model		experiment	model

type 1	*a*_1_ = 1.5 ± 0.1 nm	*a*_1_ = 1.47 nm		*D*_1_ = 0.46 ± 0.2 nm^−2^	*D*_1_ = 0.46 nm^−2^
	*b*_1_ = 1.5 ± 0.1 nm	*b*_1_ = 1.47 nm			
	θ_1_ = 88 ± 1°	θ_1_ = 90°			
type 2	*a*_2_ = 1.5 ± 0.1 nm	*a*_2_ = 1.62 nm		*D*_2_ = 0.46 ± 0.2 nm^−2^	*D*_2_ = 0.48 nm^−2^
	*b*_2_ = 1.5 ± 0.1 nm	*b*_2_ = 1.32 nm			
	θ_2_ = 82 ± 1°	θ_2_ = 85.2°			

### C343 islands formed on NiO(001)

**Figure 4:**
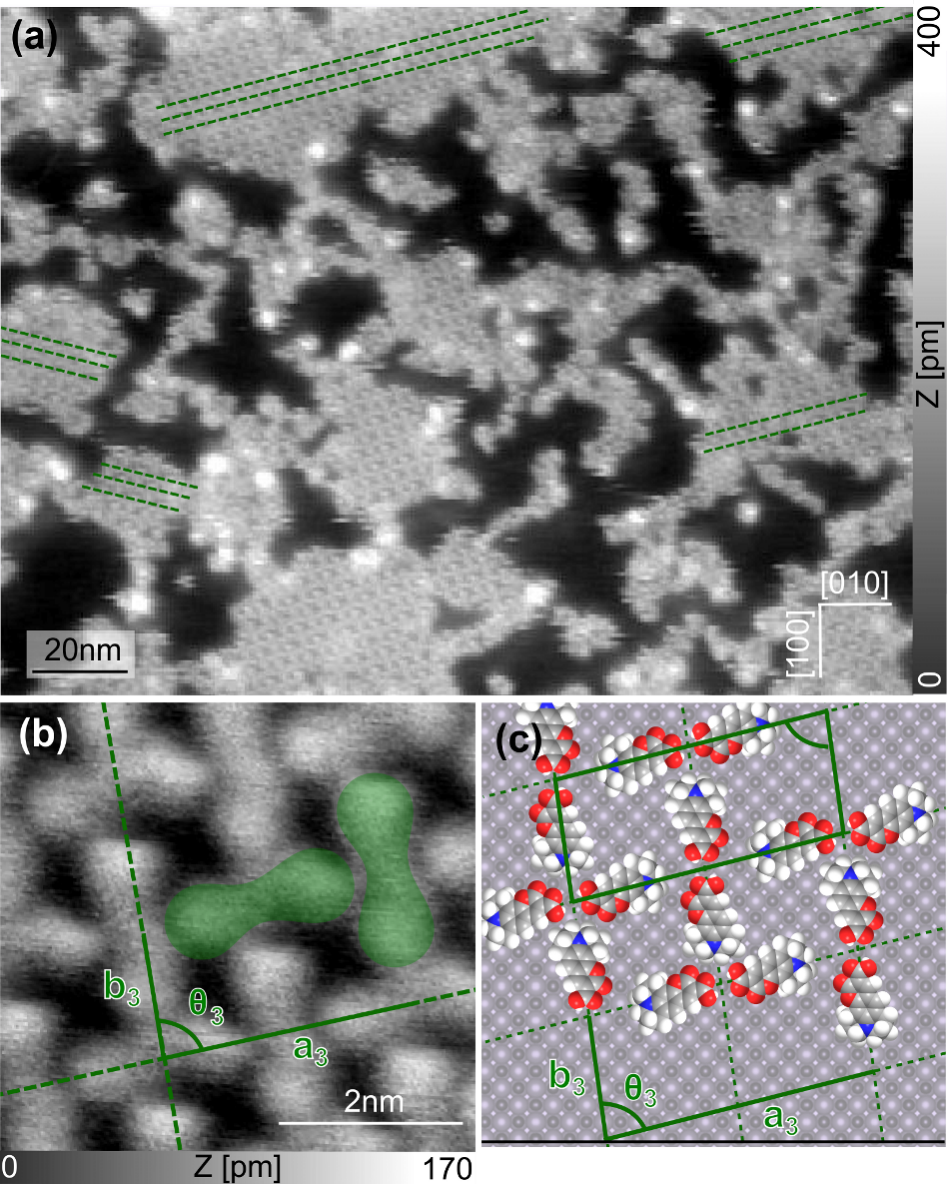
(a) Large-scale topographic image showing that C343 molecules form islands on the surface of NiO(001). (b) Close-up topographic image taken on top of one of these islands. One unit cell corresponds to four molecules arranged in two different pairs of molecules shown in green. (c) Corresponding model reproducing the mesh motive (scan parameters: 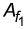 = 7 nm, Δ*f*_1_ = −7 Hz).

In a second experiment, the absorption of C343 on a clean NiO(001) surface was studied. In the large-scale topographic image shown in Figure 4a, it can be seen that C343 also forms molecular islands on the clean terraces of NiO with a typical size of 20–40 nm. The height of the C343 islands (250–300 pm) is comparable to the height of Cu-TCPP islands suggesting, again, flat lying adsorbed dye molecules. In contrast to the Cu-TCPP islands, the molecular rows are aligned only along one angle ±(15 ± 1°) with respect to the [010] direction. More precise information, including the mesh parameters *a*_3_ and *b*_3_ and the angle θ_3_ between these vectors can be determined from the close-up high-resolution nc-AFM topographic image shown in Figure 4b. In this image it can be seen (in green) that the mesh motive is composed of two different pairs of dyes, implying that a unit cell is composed of four molecules. The first pair of molecules has the molecular axis aligned along vector *a*_3_, whereas the second pair is oriented along *b*_3_ and an angle θ_3_ of 86 ± 1° is measured between these vectors. The lattice parameters *a*_3_ and *b*_3_ lie in the range of 3.5 ± 0.1 nm and 1.5 ± 0.1 nm, respectively and the molecular density is measured to be *D*_3_ = 0.74 ± 0.2 nm^−2^.

The model depicted in Figure 4c reproduces the mesh motive observed in the topographic image. It can clearly be seen that the pairs are composed of two molecules facing each other, stabilized via H-bonds between the carboxylic acid groups. This model delivers values for the mesh parameters that are compared to the experimental results in Table 2. The data demonstrate that the parameters correspond nicely, highlighting the accuracy of the model.

**Table 2:** Experimental and model parameters, respectively measured and calculated on C343 islands.

mesh parameters			molecular density	
		
experiment	model		experiment	model

*a*_3_ = 3.5 ± 0.1 nm	*a*_3_ = 3.44 nm		*D*_3_ = 0.74 ± 0.2 nm^−2^	*D*_3_ = 0.79 nm^−2^
*b*_3_ = 1.5 ± 0.1 nm	*b*_3_ = 1.47 nm			
θ_3_ = 86 ± 1°	θ_3_ = 84.1°			

### Charge-transfer direction studied by KPFM

**Figure 5:**
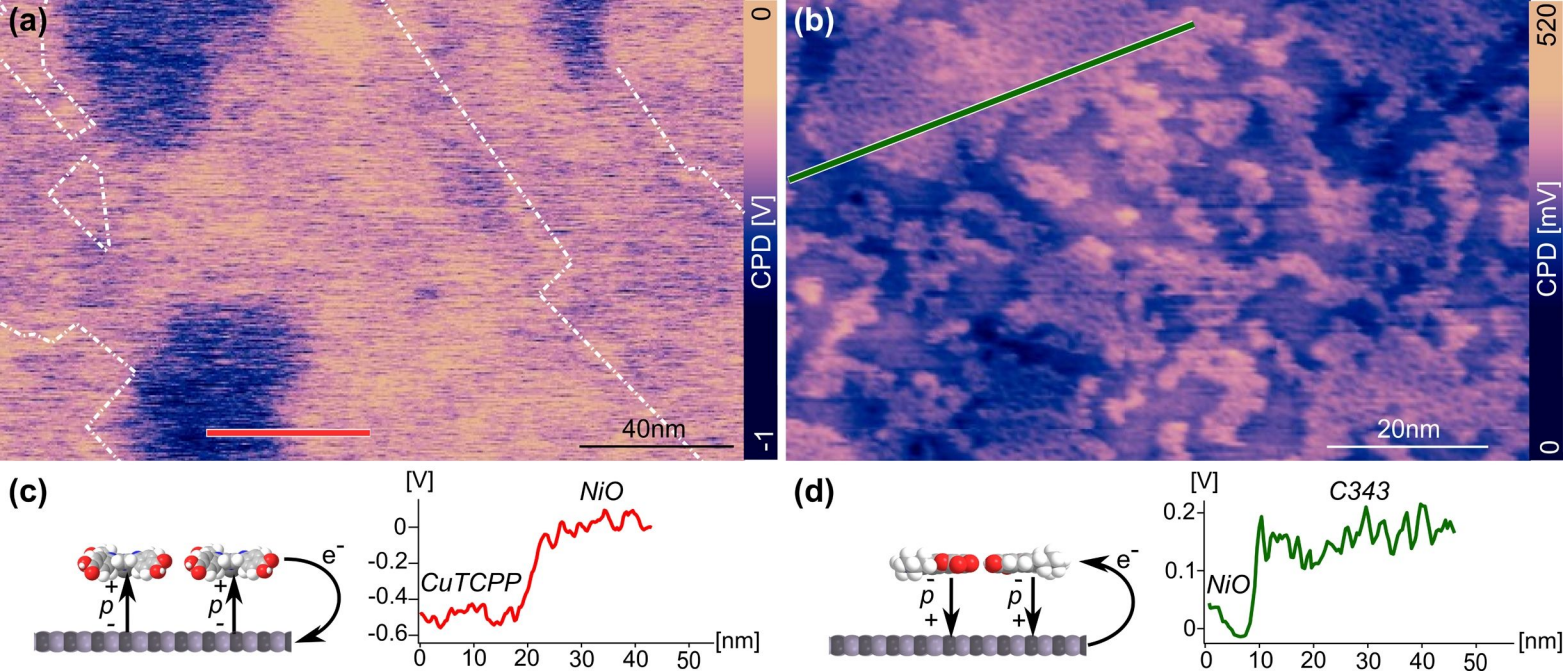
(a, b) CPD measurements of Cu-TCCP and C343 islands on the NiO(001) substrate, respectively (scan parameters: 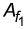 = 7 nm; Δ*f*_1_ = −7 Hz, *V*_ac_ = 800 mV, and *f*_ac_ = 1 kHz or *f*_ac_ = 250 Hz, respectively). (c, d) Profiles recorded in (a) and (b), respectively, and models highlighting the direction of the dipole moment *p* and the corresponding charge transfer.

KPFM is an analytical method that can be applied to examine the change of the work function induced by the adsorption of organometallic complexes on surfaces at the nanoscale. Using this method, the CPD between the surface of NiO and the different molecular islands was measured. Depending on the tip, it has been observed, that the absolute CPD values recorded on NiO can vary by roughly ±100 mV. Therefore, in order to facilitate the comparison between Cu-TCPP and C343, the CPD of NiO was set as reference (0 V) and all CPD values given below are relative values. The values of the CPD between the surface and the molecular islands measured by using KPFM [25] are given in Figure 5a and Figure 5b. These large-scale KPFM images were acquired simultaneously with the topographic images presented in Figure 3a and Figure 4a, respectively.

In Figure 5a, as well as in the profile recorded along the red line present in this image and displayed in Figure 5c, it can be seen that the CPD, and hence the work function, is decreased above the islands in comparison to the surface of NiO. This effect can be related to a more positively charged island compared to the substrate. This is attributed to the creation of a surface dipole moment *p* (see Figure 5c). In the present case, the positive end of the dipole moment is pointing towards the molecular layer. Consequently, this results in an electron transfer from the molecules to the surface of NiO, which is expected for a dye such as Cu-TCPP originally designed for an n-type semiconductor. The value of the dipole moment as well as the partial charge transfer can be calculated from the measured CPD values [37–38] (see Supporting Information File 1). On type-1 and type-2 islands, the average CPD difference between the molecular layer and the surface is Δ*V*_CPD_ = −400 ± 50 mV. Considering a flat-lying adsorption geometry of the molecules and knowing that the molecular densities of both types of islands are in the same range, this is attributed to an average dipole moment of −2.2 D/molecule independent of the island type. This, in turn, corresponds to a partial charge transfer of +0.35*e*^−^/molecule. Calculated as a function of the active area, this corresponds to a value +0.16*e*^−^/nm^2^.

The adsorption KPFM measurement of C343, which is designed to be implemented in a p-type device, is shown in Figure 5b. In this image it can be seen that, at a large scale, the CPD contrast is slightly modulated. This is attributed to variations of surface charges resulting from the cleavage process. To get a clear and unambiguous CPD contrast one has to focus on smaller areas where long-range charge variations do not interfere with the determination of the relative CPD between NiO and molecular islands (see Supporting Information File 1). Nevertheless, Figure 5b, as well as the profile recorded along the green line and displayed in Figure 5d, show that the CPD, and therefore also the work function, is locally increased above the molecular layer compared to the surface of NiO. Thus, in contrast to Cu-TCPP, the electron transfer occurs from the substrate towards the molecules (see schematic in Figure 5d). The average CPD change between the islands and the surface is measured to be Δ*V*_CPD_ = +150 ± 30 mV. Based on a flat-lying adsorption geometry of the molecules, an average dipole moment of +0.5 D/molecule is calculated. This corresponds to a partial charge transfer of −0.08*e*^−^/molecule occurring in the opposite direction to Cu-TCPP, and that C343 is compatible with application in a p-type device. Furthermore, this also implies that, in terms of charge transfer, C343 is about four times less efficient than Cu-TCPP. However, because C343 has a larger molecular density than Cu-TCCP, the charge transfer intensity appears to be roughly equal to −0.06*e*^−^/nm^2^ when calculated as a function of active area instead of single molecules. Consequently, if we think about building a DSSC device, this implies that C343 will result in active electrodes that would be 2.5-times less efficient in terms of charge injection compared to Cu-TCPP.

To summarize the results, a comparison of Cu-TCPP and C343 is given in Table 3.

**Table 3:** Comparison between Cu-TCPP and C343 adsorbed on NiO(001).

molecules	Δ*V*_CPD_	dipole moment		partial charge transfer	
		
		molecule^−1^		molecule^−1^	nm^−2^

Cu-TCPP	−400 ± 50 mV	−2.2 D		+0.35*e*^−^	+0.16*e*^−^
C343	+150 ± 30 mV	+0.5 D		−0.08*e*^−^	−0.06*e*^−^

## Conclusion

The adsorption of Cu-TCPP molecules on the surface of NiO(001) was investigated by nc-AFM and compared to that of C343 molecules. Using high-resolution topographic measurements, it was shown that both molecules lie flat on the surface and form islands. Different types of islands, where molecules are aligned with different angles with respect to the crystallographic directions of the surface, are observed. Using these topographic measurements as well as appropriate models reproducing accurately the mesh motives, the molecular densities from both molecules could be estimated. By combining these results with KPFM measurements, the average dipole moment of both molecular assemblies were determined. Comparing the two molecules adsorbed on NiO, their charge transfer directions are found to be opposite: Cu-TCPP is observed to be an electron acceptor whereas C343 is an electron donor, meaning that the latter is effectively more suitable for the design of p-type DSSCs. However, it has also been shown that, active areas composed of Cu-TCPP molecules are about 2.5-times more efficient in terms of charge transfer compared to C343 domains (+0.16*e*^−^/nm^2^ vs −0.06*e*^−^/nm^2^).

## Experimental

### Sample preparation

The NiO(001) crystals used in this study were purchased from SurfaceNet. They consist of a rectangular rod with dimensions 2 × 2 × 7 mm^3^ and a long axis in the [001] direction. The NiO(001) surface was prepared by in situ cleavage with prior and subsequent annealing (at 600 °C and 500 °C, respectively) resulting in an atomically clean surface. Molecules were then thermally evaporated, from commercially available molecular powders, at RT and under UHV conditions (*p <* 1 × 10^−10^ mbar) on the clean surface of NiO. Different deposition parameters were used for Cu-TPP and C343. **Cu-TCPP: ***T*_evaporation_ = 315 °C, *t*_deposition_ = 5 min and a rate of 0.5 Å/min; **C343: ***T*_evaporation_ = 150 °C *t*_deposition_ = 5 min and a rate of 0.5 Å/min. After C343 deposition, the sample was annealed for 30 min at 80 °C.

### Scanning probe microscopy

All measurements were carried out in dark using a custom-built atomic force microscope operating under UHV at RT. All AFM images were recorded in the non-contact mode, using silicon cantilevers (Nanosensors PPP-NCL, stiffness *k* = 20–30 N/m, resonance frequency *f*_1_ ≈ 165 kHz, quality factor *Q_f1_* ≈ 30000) with compensated contact potential difference. Kelvin probe force microscopy was performed in frequency-modulation mode using a voltage modulation applied together with the dc compensation voltage to the sample (*V*_ac_ = 800 mV and *f*_ac_ = 1 kHz or 250 Hz).

## Supporting Information

The Supporting Information discusses the determination method of the average CPD difference and shows that the CPD can be determined locally when C343 is adsorbed on NiO.

File 1 Additional experimental data.
