# Supplementary material for: Comparing a porphyrin- and a coumarin-based dye adsorbed on NiO(001)
Source: Beilstein J Nanotechnol. 2019 Apr 15;10:874–81. doi: 10.3762/bjnano.10.88 (PMC6541355; doi:10.3762/bjnano.10.88)
Supplement: File 1 — Additional experimental data. [file Beilstein_J_Nanotechnol-10-874-s001.pdf]

BEILSTEIN JOURNAL OF NANOTECHNOLOGY

Supporting Information

for

Comparing a porphyrin- and a coumarin-based dye adsorbed on NiO(001)

Sara Freund, Antoine Hinaut, Nathalie Marinakis, Edwin C. Constable, Ernst Meyer, Catherine E. Housecroft and Thilo Glatzel

Beilstein J. Nanotechnol. **2019,** *10,* 874–881. doi:10.3762/bjnano.10.88

Additional experimental data

License and Terms: This is a supporting information file under the terms of the Creative Commons Attribution License (http://creativecommons.org/licenses/by/4.0). Please note that the reuse, redistribution and reproduction in particular requires that the authors and source are credited.
The license is subject to the *Beilstein Journal of Nanotechnology* terms and conditions: (https://www.beilstein-journals.org/bjnano)

Determination of the average CPD difference

This section describes how the average CPD difference ($\Delta_{CPD} = CPD_{island} - CPD_{NiO}$) was measured and how the resulting partial charge transfer was calculated. In a first step two different masks were drawn on the topographic images. The first one (in red) over the NiO substrate and the second (in blue) over the molecular islands (Figure S1a,b). Island edges were excluded to avoid edge effects that can strongly influence the measured CPD value.

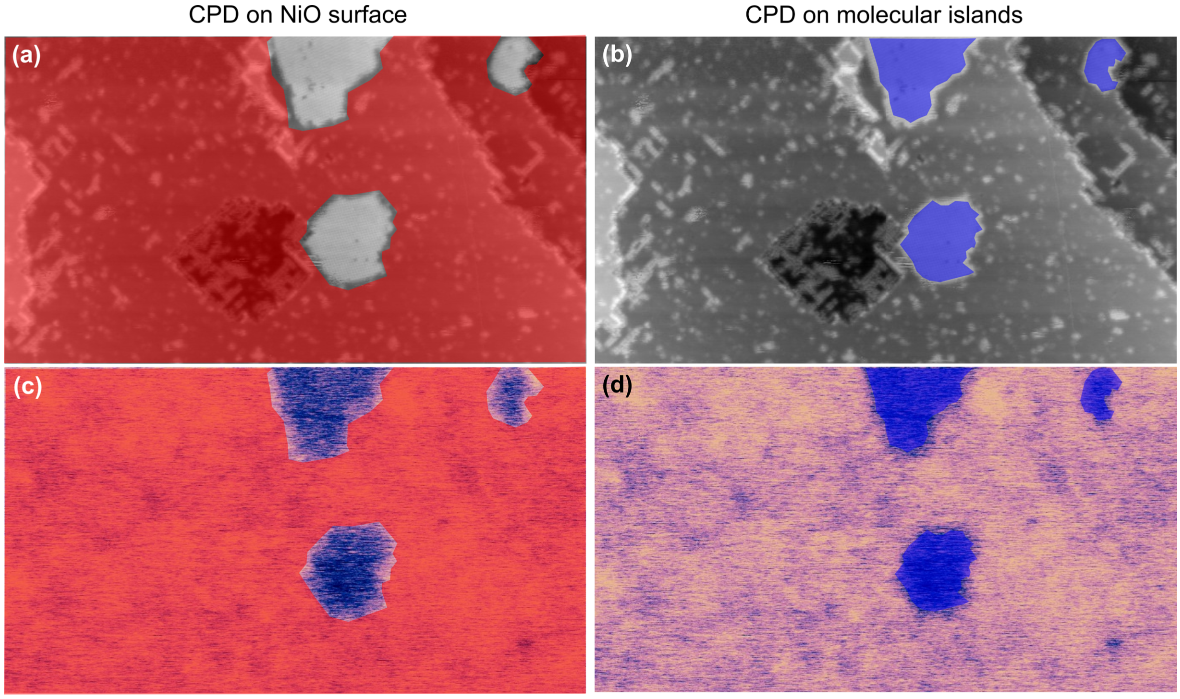

Figure S1: Determination of the average CPD. (a, b) nc-AFM topographic images superimposed with a mask on the bare NiO substrate (red) and on the molecular islands (blue), respectively. (c, d) KPFM images superimposed with corresponding masks. Scan parameters: $A_{f_1} = 4$ nm; $\Delta f_1 = -13$ Hz, $\omega = 900$ Hz and V_{ac} = 800 mV.

In a second step, these two masks were superimposed to the corresponding KPFM images (Figure S1c,d) and the average CPD value was measured in these masked areas. This process was repeated on several images for both molecules, Cu-TCPP and C343, to lower the error range. Since CPD strongly depends on the tip, its value was varying from one image to the other but the CPD difference was constant: $\Delta_{CPD} = 400 \pm 50$ mV for the Cu-TCPP island and $\Delta_{CPD} = 150 \pm 30$ mV for the C343 layers.

The difference in dipole moment densities (Δp) can be calculated by the following formula [1,2]:

$$\Delta V_{CPD} = V_{CPD}^{island} - V_{CPD}^{NiO} = 1/\varepsilon_0 \cdot \Delta p.$$

Knowing the molecular density, which could be calculated from high-resolution images, the average dipole moment can be estimated. Assuming that the effective surface–molecule distance corresponds to half of the measured island height (ca. 130 pm), the partial charge transfer between the molecules and the surface that is attributed to this dipole moment can be calculated.

Local CPD on C343 island

In our work we faced the problem that the surface of NiO(001) can be charged after cleavage. This can result in an ambiguous Kelvin contrast, which makes the determination of the average CPD difference value more difficult. Nevertheless, Figure S2 shows that the relative CPD can be easily determined locally in areas where surface charges play only a minor role. Figure S2a represents a topographic image of C343 island on the surface of NiO(001). In this image, different areas showing island edges and bare NiO can be selected. The corresponding CPD measurements are shown in Figure S2b–e. It can be seen that the charge transfer always occurs in the same direction (from the NiO surface to the molecular islands).

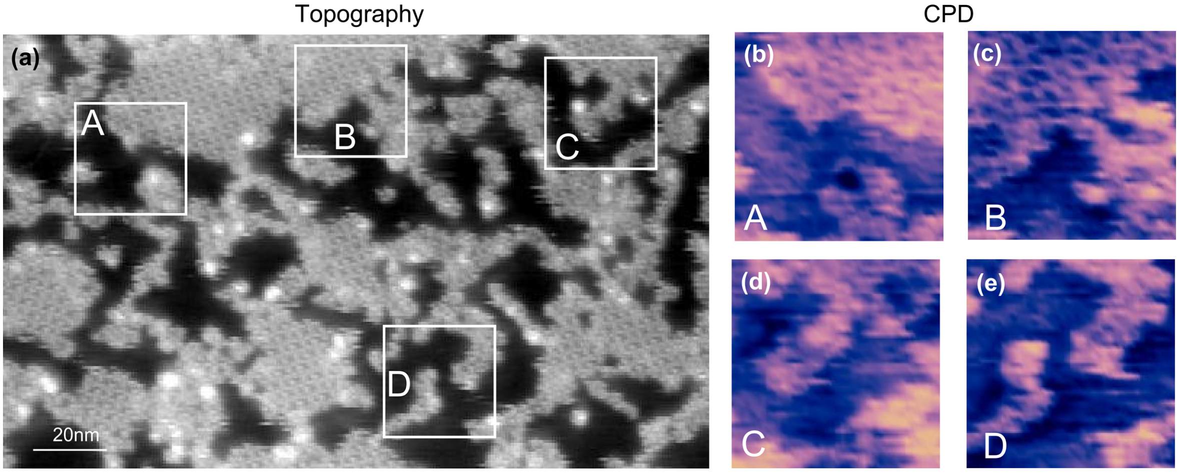

Figure S2: Determination of the average CPD. (a) nc-AFM topographic images of C343 islands adsorbed on NiO(001). (b–e) KPFM images showing that the CPD can be determined locally in the areas A, B, C and D marked in (a). Scan parameters: $A_{f_1} = 7$ nm; $\Delta f_1 = -7$ Hz; $\omega = 250$ Hz; $V_{ac} = 800$ mV.

References

1. Milde, P.; Zerweck, U.; Eng, L. M.; Abel, M.; Giovanelli, L.; Nony, L.; Mossoyan, M.; Porte, L.; Loppacher, C. *Nanotechnology* **2008**, *19*, 305501. doi:10.1088/0957-4484/19/30/305501.

2. Glatzel, T.; Zimmerli, L.; Koch, S.; Kawai, S.; Meyer, E. *Appl. Phys. Lett.* **2009**, *94*, 063303. doi:10.1063/1.3080614.
